# Supplementary material for: Effectiveness and Safety of Ayurvedic Medicines in Type 2 Diabetes Mellitus Management: A Systematic Review and Meta-Analysis
Source: Front Pharmacol. 2022 Jun 8;13:821810. doi: 10.3389/fphar.2022.821810 (PMC9213670; doi:10.3389/fphar.2022.821810)
Supplement: Supplementary file 1 [file DataSheet1.zip › Supplementary Material/Forest and Funnel Plots/Gynostemma pentaphyllum/FBG.pdf]

Gynostemma pentaphyllum - FBG

Study	Intervention N	Intervention Mean	Intervention SD	Comparator N	Comparator Mean	Comparator SD	Mean Diff. with 95% CI	Weight (%)
Huyen 2010	12	-54.05	32.43	12	-10.81	39.64	-43.24 [-72.22, -14.26]	21.14
Huyen 2012	12	120.71	7.21	13	145.93	18.02	-25.22 [-36.16, -14.28]	78.86
**Overall**							-29.03 [-43.45, -14.61]	

Heterogeneity: $\tau^2 = 37.50$, $I^2 = 23.10\%$, $H^2 = 1.30$

Test of $\theta_i = \theta_j$: Q(1) = 1.30, p = 0.25

Test of $\theta = 0$: z = -3.95, p = 0.00

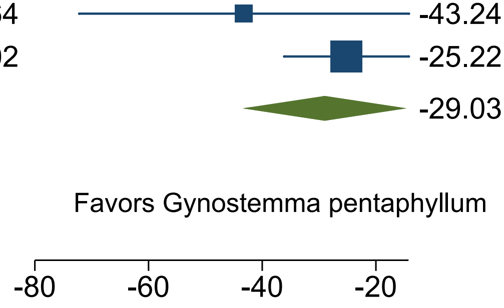

Random-effects REML model
